# Mechanism of LncRNA Gm2044 in germ cell development

**DOI:** 10.3389/fcell.2024.1410914

**Published:** 2024-07-04

**Authors:** Qinran Zhu, Junpei Sun, Chuangchuang An, Xin Li, Shumin Xu, Yutong He, Xinyi Zhang, Lei Liu, Ke Hu, Meng Liang

**Affiliations:** ^1^ School of Life Science, Bengbu Medical University, Bengbu, China; ^2^ First Affiliated Hospital, Bengbu Medical University, Bengbu, China

**Keywords:** lncRNA, spermatogenesis, estrogen, ovaries, miRNA

## Abstract

Germ cell development in mammals is a complex physiological process that involves the proliferation of primordial germ cells, meiosis, and the formation of male and female gametes. Long non-coding RNA (lncRNA) is a type of RNA with more than 200 nucleotides that does not code for proteins. A small number of lncRNAs have been shown to participate in spermatogenesis in the testes and in follicular development in the ovaries, but the role of the vast majority of lncRNAs and their molecular mechanisms still need further study. LncRNA Gm2044 was identified as a differentially expressed lncRNA in mouse spermatogenesis by microarray technology. In mouse testis, lncRNA Gm2044 can act as competing endogenous RNA to regulate SYCP1 expression in GC-2 cells derived from mouse spermatocyte cells, and it can also act as a host gene for miR-202 to regulate RBFOX2 protein expression. In female mouse ovaries, lncRNA Gm2044 regulates 17β-estradiol synthesis through the miRNA-138-5p-Nr5a1 pathway or by interacting with EEF2. In addition, studies suggest that lncRNA Gm2044 is also involved in the progression of reproductive system diseases such as male nonobstructive azoospermia. Here, we summarize the roles and molecular mechanisms of lncRNA Gm2044 in male and female gametogenesis and its potential role in some infertility disorders.

## 1 Introduction

The development of mammalian germ cells is a complex and delicate physiological process, which mainly includes spermatogenesis in the male testis and oocyte generation in the female ovary. Mammalian spermatogenesis needs to go through three stages: Self-renewal and mitosis of spermatogonial stem cells (SSC), meiosis of spermatocytes, and differentiation of haploid spermatids, and eventually the production of mature sperm (Kubota and Brinster, 2018). The development of oocytes begins in the fetal ovary and matures in adulthood through meiosis. Such a long period of meiotic arrest makes it easy for the oocytes to be affected by the surrounding microenvironment. The surrounding somatic cells of oocytes can regulate the growth and development of oocytes by regulating hormones, growth factors, and metabolites (Petro et al., 2012). However, the role of the ovary is not only to produce functional oocytes, but also to synthesize and secrete some of the steroid hormones necessary for fertilization and germ cell development, such as estrogen, progesterone, and androgen (Palermo, 2007; Alexander et al., 2023). These steroid hormones are all produced by cholesterol in mitochondria through complex biosynthetic pathways (Miller, 2013; Esmaeilian et al., 2023). Estradiol is an endogenous hormone secreted by mature ovarian follicles (Yang et al., 2023), and changes in its level not only play a key role in the regulation of female reproductive development, but also in the development of mammalian bones, brain function and skin health (Baddela et al., 2023).

It is well known that lncRNA is a class of non-coding RNA molecule with a length of more than 200 nucleotides. In the early years, there was a lack of research and understanding of lncRNA. Three-quarters of the human genome can be transcribed into RNA, but about 99% of these mRNAs cannot encode proteins (Wu et al., 2020). As a result, many people believe that lncRNAs are just “junk” or “transcriptional noise” generated by gene transcription, and the importance of lncRNA has not been apparent until recent years (Palazzo and Lee, 2015). Research has shown that lncRNA can be expressed through differences involved in the pathogenesis of diseases such as inflammation, metabolic diseases, and cancer. For example, in inflammation, lncRNA TNF can participate in the progression of non-alcoholic steatohepatitis (NASH) (Atanasovska et al., 2021), and lncRNA Helf promotes hepatic inflammation and fibrosis (Han et al., 2023). In metabolic diseases, lncRNA TUG1 can improve diabetic nephropathy (Meng et al., 2022), and LncRNA H19 is involved in osteoblast differentiation (Liang et al., 2016). There are also many lncRNAs involved in cancer, and lncRNA HOTTP is the most classic. LncRNA HOTTIP can play an important role in the pathogenesis of various cancers. Moreover, the knockdown of HOTTIP can inhibit the proliferation of gastric cancer cells (Xiao et al., 2020), and patients with high HOTTIP expression have higher metastasis potential of rectal cancer cells (Rui et al., 2019). HOTTIP is also highly expressed in ovarian cancer cells and can promote the development of ovarian cancer and metastasis of ovarian cancer cells by regulating the transcription factor HIF-1α or pyroptosis (Tan et al., 2021; Zhang et al., 2022). Increased lncRNA HOTTIP can regulate γ-H_2_AX and p53 signaling in UV-induced spermatogonia G2/M arrest and early apoptosis (Liang and Hu, 2019). In addition, our previous study revealed that hundreds of significantly differentially expressed lncRNAs were identified in testicular heat exposure mouse by high-throughput sequencing, and these lncRNAs may act synergistically with associated differentially expressed cirRNAs, miRNAs, and mRNAs (Hu et al., 2021). More and more studies have confirmed that lncRNA can participate in various biological processes.

In recent years, the effects of lncRNA on reproductive cells has been reported (He et al., 2021). Premature ovarian insufficiency (POI) is a disease of the female reproductive system. LncRNA HCP5 is involved in the progression of POI by regulating the expression of two genes, *MSH5* and *YB1* (Wang et al., 2020). The high expression of lncRNA PCAT6 can inhibit the expression level of PTEN, thereby promoting the development of ovarian cancer (Kong et al., 2019). Overexpression and knockdown of lncRNA5251 can decrease and improve sperm quality in mice, respectively, and lncRNA5251 can regulate spermatogenesis through interaction with cell link-related genes (Zhang et al., 2023). An increasing number of non-protein-coding RNAs have been confirmed to be involved in the regulation of mammalian germ cell genesis. Among these, lncRNA Gm2044 is specifically expressed in the ovaries and testis of mice and has regulatory effects on the processes of estrogen synthesis and spermatogenesis through related signaling pathways. This paper describes the important roles and molecular mechanisms of lncRNA Gm2044 in the testis and ovaries.

## 2 LncRNA Gm2044

### 2.1 Classification of lncRNA

The complex molecular mechanism of lncRNA production has been widely reported. The most common classification divides lncRNAs into three categories according to their position in the genome: 1) Intronic lncRNAs, which are transcribed from the intron region of coding genes, e.g., Mrhl lncRNA, which is transcribed from the 15th intron of mouse *Phkb* gene on chromosome 18, was found to regulate the meiosis of B-type spermatogonia in mouse testis by regulating related signaling pathways or mediating *Sox8* (Kataruka et al., 2017; Kayyar et al., 2023). 2) Sense and antisense lncRNAs, which are transcribed from either the sense or antisense strand of the coding gene, e.g., lncRNA NNT-AS1 is an antisense lncRNA that can promote the progression of esophageal squamous cell carcinoma by acting as a sponge for miR-382-5p (Pan et al., 2022). 3) Intergenic lncRNA (lincRNA) is transcribed in the middle region of two coding genes (Ma et al., 2013) e.g., the recently discovered two lincRNAs, which have a significant function in mouse macrophage inflammatory regulation (lincRNA-Cox2) (Salih et al., 2023) and play an important role in cancer cells (lincRNA p21) (Huang et al., 2022).

### 2.2 Discovery of lncRNA Gm2044

**FIGURE 1:**
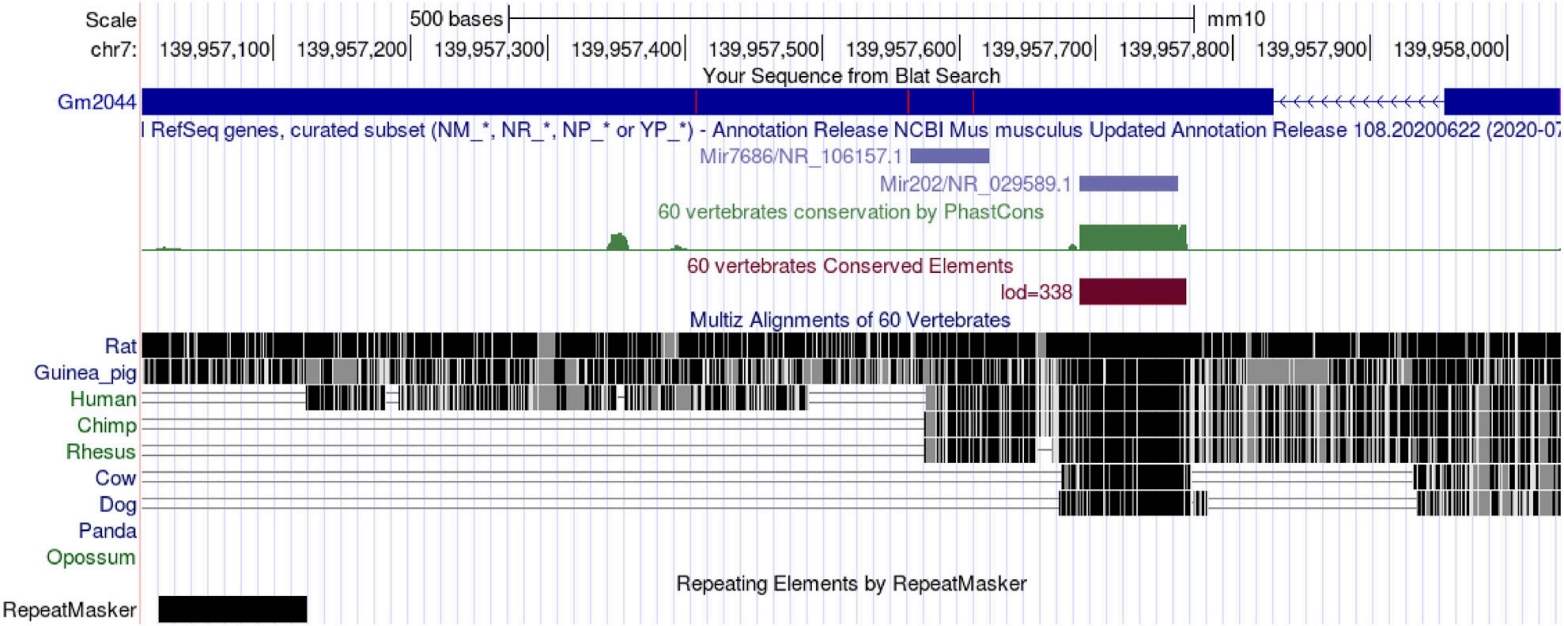
LncRNA Gm2044 is located on mouse chromosome seven and is the host gene of miR-202. Moreover, lncRNA Gm2044 is highly conserved in rodents.

We know that sperm originate from primordial germ cells (PGCs), and PGC is produced by embryonic cells that have been partially converted into somatic cells (Ohinata et al., 2009; Stukenborg et al., 2014). PGC produces SSCs through a complex process (Phillips et al., 2010). SSC can both self-renew and differentiate into spermatogonia (SPG), which can be divided into type A spermatogonia (A), Intermediate (In), and type B spermatogonia (B) (Subash and Kumar, 2021). Type B spermatogonia undergo mitosis for proliferation or meiosis to transform into primary spermatocytes, and the primary spermatocytes undergo meiosis to differentiate into the secondary spermatocytes, resulting in a haploid round sperm (RS) that becomes a mature elongated sperm (Stukenborg et al., 2014; Cannarella et al., 2020). In addition, Sertoli cells are required to provide nutrition and support, and Leydig cells secrete androgens to regulate spermatogenesis precisely (Zhou et al., 2019). But the function of lncRNA in mammalian reproductive cells remains largely unexplored. So some groups have analyzed the expression profiling of lncRNAs in germ cells at different stages of the testis by microarray (Bao et al., 2013; Sun and Wu, 2015). Liang et al. analyzed the expression of lncRNA and mRNAs in four specific types of germ cells in the testis by microarray analysis (Liang et al., 2014). The four types of germ cells were spermatogonial stem cells, type A spermatogonia, pachytene spermatocytes (PS), and round spermatocytes. The expression of lncRNA was the highest in type A spermatogonia and the lowest in pachytene spermatocytes, while the expression trend of mRNA was similar to that of lncRNA, indicating that lncRNA and mRNA may play a synergistic role in spermatogenesis (Liang et al., 2014). Further analysis identified four lncRNAs that were differentially expressed in male germ cells. The UCSC genome Browser database (https://genome.ucsc.edu/) showed that lncRNA Gm2044 was 912 nt in length, had two exons, and was located on chromosome 7qF4 (Figure 1). In addition, four ORFs were predicted in ORF Finder (https://www.ncbi.nlm.nih.gov/orffinder/): ORF4 could not translate proteins on the lncRNA antisense chain, and the other three ORFs were verified by experiments and could not translate proteins, so lncRNA Gm2044 was proved to be non-coding RNA (Hu et al., 2022).

## 3 Regulatory mechanism of lncRNA Gm2044 in male reproduction

### 3.1 LncRNA Gm2044 interacts with mRNA

To understand the role of lncRNA Gm2044 in germ cells, the potential effect of lncRNA Gm2044 on spermatogenesis in the testis was first investigated. In our previous study, we found that lncRNA Gm2044 was highly expressed in pachytene spermatocytes and meiosis of the mouse testis by microarray analysis (Liang et al., 2014). And its expression level was also downregulated when the number of spermatocytes was relatively low due to the differentiation of spermatocytes into spermatides (Hu et al., 2018). This shows that lncRNA Gm2044 is important in the meiosis of spermatogenesis. UTF1, or undifferentiated embryonic cell transcription factor 1, is expressed in early embryonic cells and primordial germ cells of mammals and has the function of regulating gene expression and cell proliferation and differentiation (Okuda et al., 1998; Raina et al., 2021). In addition, UTF1 has an effect on male germ cells. Inactivation of UTF1 leads to a decrease in the number of germ cells at birth, while conditional inactivation of UTF1 leads to impaired spermatogenesis in adult mice, indicating that UTF1 plays an important role in spermatogenesis (Kasowitz et al., 2017). LncRNA Gm2044 is located on the minus strand of DNA, while *Utf1* is located on its adjacent plus strand. Blast analysis showed that *Utf1* mRNA and lncRNA Gm2044 had five complementary sequences (Hu et al., 2018). Furthermore, overexpression of lncRNA Gm2044 in mouse spermatogonia and knockdown of lncRNA Gm2044 in mouse spermatocytes resulted in the inhibition and increase in UTF1 translation level, respectively, but did not affect *Utf1* mRNA. This suggests that lncRNA Gm2044 can regulate the expression of UTF1 protein by interacting with mRNA, which is important for germ cell meiosis and development (Hu et al., 2018). In addition, overexpression of lncRNA Gm2044 also reduced *Rbfox2* mRNA levels and inhibited the translation of RBFOX2 (RNA binding fox-1 homolog 2), as detailed below (Chen et al., 2017; Liang et al., 2019).

### 3.2 lncRNA Gm2044 as miRNA sponge/host gene

MiRNA is a class of endogenous non-coding RNA with regulatory functions and a length of about 20–25 nucleotides. Previous studies have demonstrated the importance of miRNAs in germ cell development (Loke et al., 2019). There are many mechanisms of interaction between miRNA and lncRNA, the most common one being that lncRNA competitively binds miRNA, that is, lncRNA acts as a miRNA sponge to regulate downstream signaling pathways and biological processes (Ma et al., 2023). MiR-202 is a regulator of meiosis initiation, and the knockdown of miR-202 can lead to premature differentiation and maturation of mouse spermatogonia (Chen et al., 2017; Chen et al., 2021). In our previous study, the expression of lncRNA Gm2044 and miR-202 was found to be significantly upregulated in non-obstructive azoospermia (NOA) with spermatogonia arrest (Liang et al., 2019). Moreover, lncRNA Gm2044 inhibited the expression of *Rbfox2* (a known direct target gene of miR-202) by acting as the host gene of miR-202 and participated in the miR-202-*Rbfox2* signaling pathway to inhibit the proliferation of human testicular embryonic carcinoma cells NCCIT (Liang et al., 2019).

**FIGURE 2:**
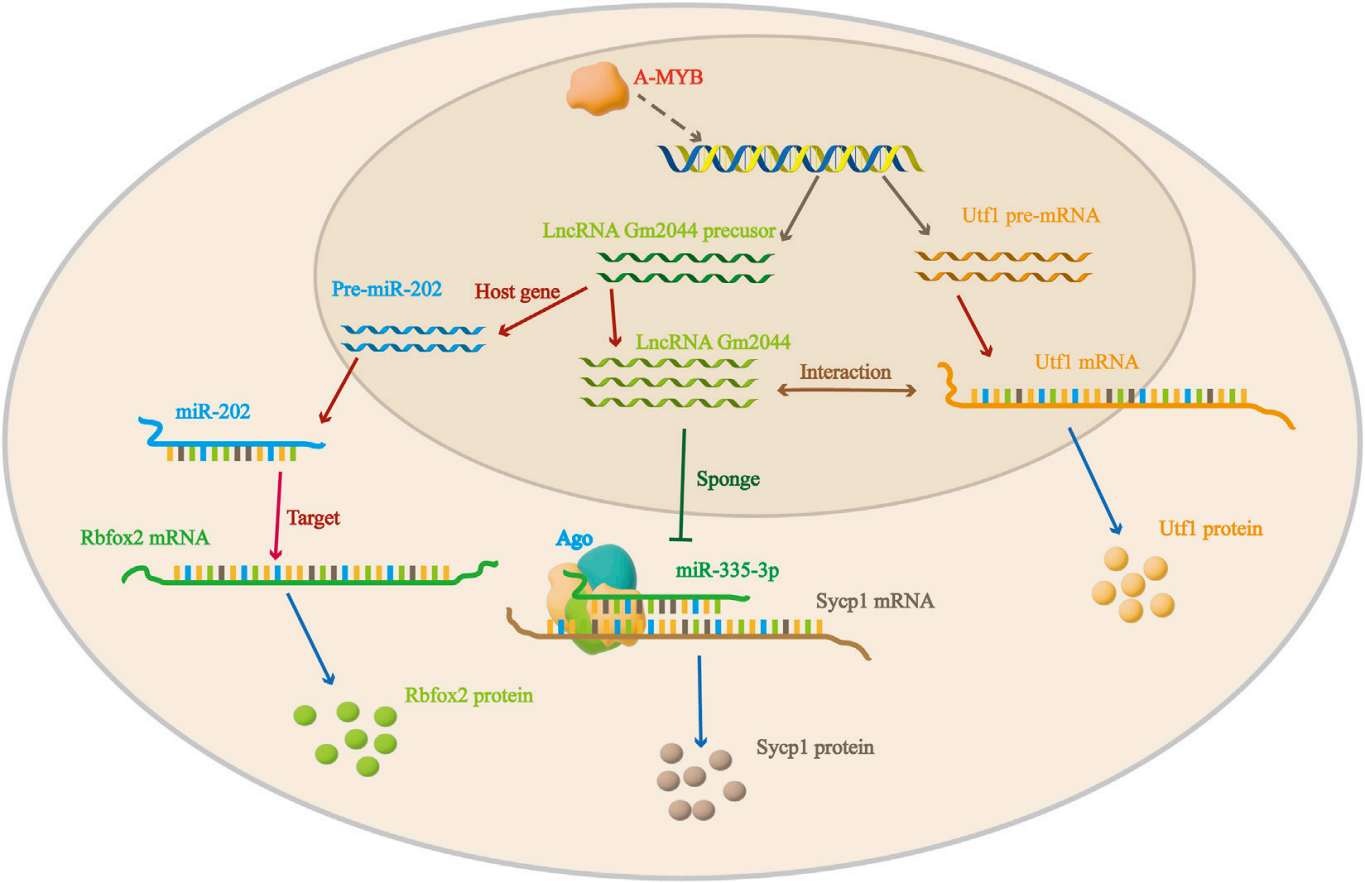
Regulation mechanism of lncRNA Gm2044 in male mouse testis.

In addition, lncRNA Gm2044 regulates the proliferation of GC-2 cells (cells differentiated from mouse spermatocytes) through its interaction with miRNA335-3P (Liang et al., 2020). A-MYB is a transcription factor encoded by the *Mybl1* gene that appears in the early stage of spermatogenesis. It can bind to the −819 bp binding site in the distal promoter region of lncRNA Gm2044 and increase the transcription level of lncRNA Gm 2044 (Tang and Goldberg, 2012; Alexander et al., 2023). LncRNA Gm2044 is a competing endogenous RNA of miRNA335-3P, which directly targets *Sycp1* (Meiotic transverse filament proteins). Overexpression of miRNA335-3P promoted SYCP1 protein synthesis (Figure 2). In summary, in the presence of A-MYB, lncRNA Gm2044 acted as a miRNA335-3P sponge to regulate the downstream signaling pathway of SYCP1 protein expression and the proliferation of GC-2 cells (Liang et al., 2020; Soheila et al., 2022). Similarly, lncRNA AK015322, which is highly expressed in spermatogonial stem cells of the testis, can act as a miR-19b-3p sponge to promote the proliferation of spermatogonial stem cells (Hu et al., 2017).

## 4 Regulation mechanism of lncRNA Gm2044 in female reproduction

### 4.1 LncRNA Gm2044 interacts with miR-138-5p to regulate estradiol synthesis

It has been reported that lncRNA plays an important role in ovarian follicle development. Through the gene annotation database BioGPS, we observed that lncRNA Gm2044 was highly expressed in pachytene spermatocytes in the testis, and it was also highly expressed in ovarian follicles, suggesting that lncRNA Gm2044 may have a potential effect on ovarian follicle development (Hu et al., 2018). RT-qRCR showed that lncRNA Gm2044 was significantly expressed in pre-antral follicular granulosa cells to preovulatory follicles in the four stages of follicular cells (Hu et al., 2019). The expression level of lncRNA Gm2044 was significantly higher in mouse pre-antral follicular granulosa cells (mpGCs) isolated from mouse follicles than in other mouse cell lines (Hu et al., 2019). LncRNA Gm2044 was predicted to be a competing endogenous RNA of miR-138-5p, and miR-138-5p has potential effects on downstream steroid hormones. *Nr5a1* was confirmed to be a direct target of miR-138-5p, and miR-138-5p inhibited the translation of *Nr5a1* mRNA and reduced the expression of NR5A1 protein (Hu et al., 2019). NR5A1, an important transcription factor for *Cyp19a1*, a key aromatase for 17β-estradiol synthesis, is a 461-amino acid nuclear receptor steroidogenic factor, and loss of *Nr5a1* causes adrenal and gonadal dysgenesis in mice. Moreover, the absence of this transcription factor in ovarian granulosa cells can lead to damage to follicle formation in the ovary, leading to infertility (Pelusi et al., 2008; Hu et al., 2019). By targeting the miR-138-5p-*Nr5a1* pathway, the inhibitory effect of knockdown of lncRNA Gm2044 on 17β-estradiol was relieved when *Nr5a1* was overexpressed in mpGCs, and *Nr5a1* upregulation promoted estradiol synthesis. This suggests that lncRNA Gm2044 can promote the synthesis of 17β-estradiol in mpGCs isolated from preantral follicles by acting as a miR-138-5p sponge (Hu et al., 2019). In addition, *Sirt1* was found to be a direct target of miR-138-5p, and lncRNA Gm2044 also promoted the synthesis of 17β-estradiol by targeting *Sirt1* with miR-138-5p (Zhu et al., 2017).

### 4.2 LncRNA Gm2044 interacts with EEF2 to regulate estradiol synthesis

**FIGURE 3:**
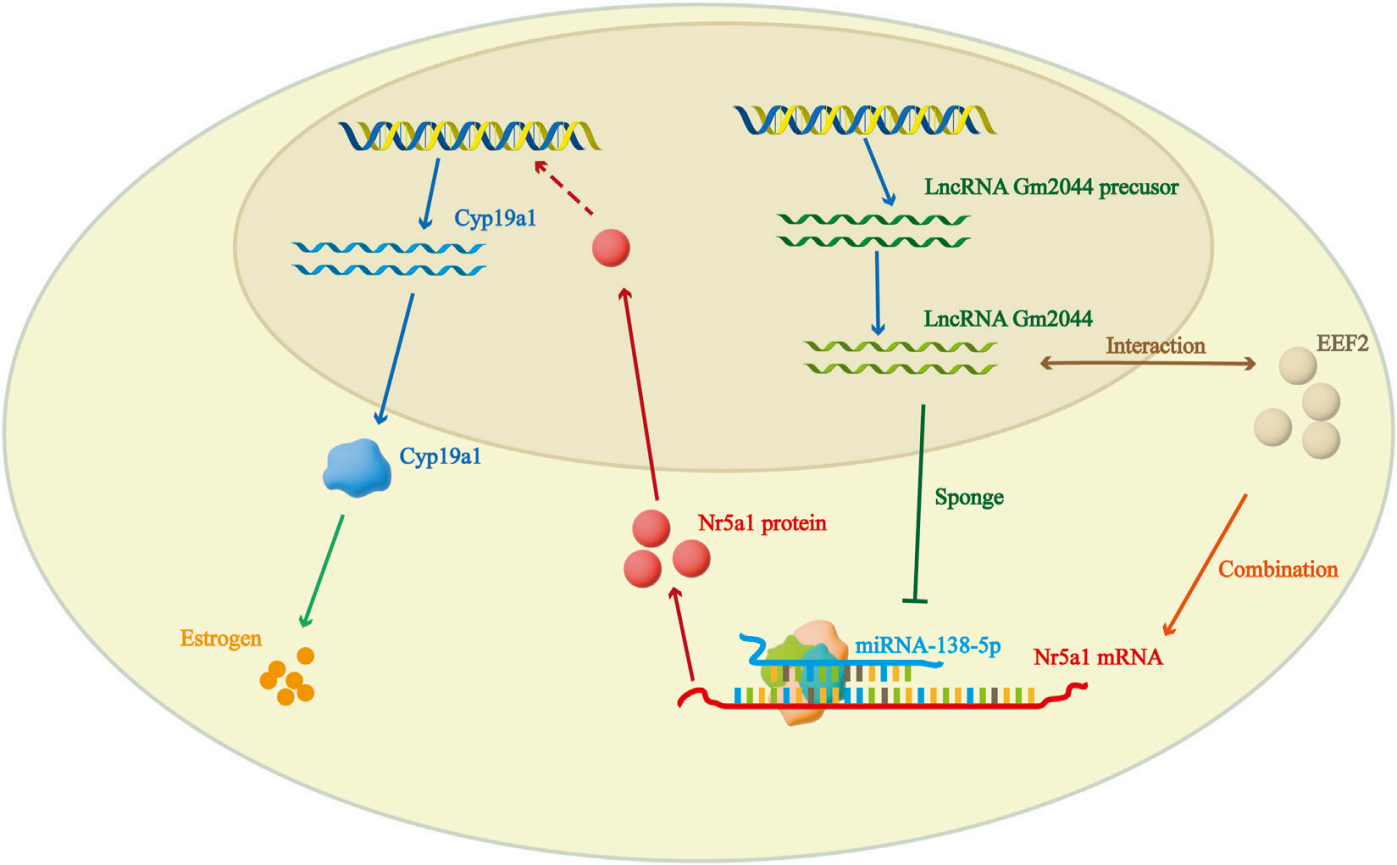
LncRNA Gm2044 can regulate 17β-estradiol synthesis by interacting with EEF2 or by acting as a miRNA sponge.

Recent studies have found that EEF2 is also involved in the regulation of 17β-estradiol by lncRNA Gm 2044. There have been many studies on EEF2, for example, miR-642a-5p can directly target EEF2 to mediate the high permeability and apoptosis of pulmonary microvascular endothelial cells induced by lipopolysaccharide (Fei et al., 2020). EEF2 plays a dual role in the apoptosis of cardiomyocytes during myocardial ischemia-reperfusion (Zhang et al., 2017). ChIRP-MS analysis of adolescent mouse ovaries showed that 21 proteins could specifically bind to lncRNA Gm 2044. Bioinformatics analysis showed that EEF2 may affect the synthesis of 17β-estradiol by interacting with lncRNA Gm 2044. To understand the mechanism of lncRNA Gm2044 and EEF2 in the female ovary, lncRNA Gm2044 knockout mice were generated. The results showed that the fertility of lncRNA Gm2044 knockout mice was similar to that of normal mice, but the concentration of estradiol was significantly reduced. Meanwhile, overexpression of lncRNA Gm2044 increased the NR5A1 protein level and estradiol concentration, while *Eef2* knockdown attenuated the effects of lncRNA Gm2044 on NR5A1 and estradiol concentration because EEF2 can bind to *Nr5a1* mRNA and promote NR5A1 protein synthesis. Finally, it promotes an increase in estradiol concentration. These results suggest that Gm2044 interacts with EEF2 to promote estradiol synthesis (Figure 3). In addition, some differentially expressed genes (*Tph1*, *Sost*, etc.) were found to be involved in estradiol synthesis in lncRNA Gm2044 KO mice, and the new regulatory network composed of these differentially expressed genes and lncRNA Gm2044 will provide a new scheme for studying estradiol in the ovary (Hu et al., 2023).

## 5 Reproductive function diseases potentially related to lncRNA Gm2044

### 5.1 Non-obstructive azoospermia

There are also many types of male infertility, and about 15% of infertile men each year suffer from azoospermia, such as NOA caused by spermatogenesis disorders and obstructive azoospermia caused by obstruction of the vas deferens, in which no sperm are found in the semen. The common causes of NOA include congenital chromosomal abnormalities, gene mutations, and varicocele, but the specific pathogenesis of NOA remains unknown (Peña et al., 2020). A previous study found that lncRNA participates in the progression of NOA (Zhou et al., 2022). LINC00467 upregulated the expression levels of genes related to spermatogenesis (*TDRD6* and *LRGUK*) to promote the progression of NOA (Bo et al., 2020). LncRNA Gm2044 was found to be highly expressed in the spermatocyte cell line GC-2, abnormally expressed in NOA with spermatogonia arrest, and able to inhibit the proliferation of mouse spermatogonia GC-1 and mouse spermatogonia GC-2 (Liang et al., 2019), suggesting that lncRNA Gm2044 is involved in the progression of NOA, which is an interesting finding. However, the specific role of lncRNA Gm2044 in the pathogenesis of NOA has not been elucidated and needs further study.

### 5.2 Polycystic ovary syndrome

It has been reported that approximately 10% of couples of reproductive age worldwide are affected by infertility, a disorder of reproductive dysfunction (Agarwal et al., 2021). There are many causes of infertility, but for women, infertility caused by ovulation disorder due to polycystic ovary syndrome (PCOS) is the most common. However, the etiology of PCOS remains unclear; it may be caused by a combination of environmental, genetic, and other diseases, and the estrogen in PCOS patients is often unbalanced. Some lncRNA are related to PCOS. For example, silencing of lncRNA UCA1 can mediate the AKT signaling pathway to inhibit the progression of PCOS (Yang et al., 2021). LncRNA BANCR is involved in PCOS by up-regulating the pro-apoptotic protein Bax and p53 to promote apoptosis (Yang et al., 2019). CYP19A1 is an aromatase that can convert steroid hormones to estrogen, and together with CYP11A1, CYP17A1 may play a key role in the progression of PCOS (Parween et al., 2020; Heidarzadehpilehrood et al., 2022). *Nr5a1* is an important transcription factor of CYP19A1 and a target gene of miR-138-5p, suggesting that lncRNA Gm2044 may regulate its downstream signaling pathway by acting as a sponge for miR-138-5p-*Nr5a1*. It may eventually participate in the pathogenesis of PCOS (Hu et al., 2019), which provides new ideas for the treatment of female infertility in the future.

## 6 Future perspectives

In recent years, more and more attention has been paid to lncRNA, and the main functional mechanisms of lncRNAs have gradually become clear. LncRNAs can be divided into those involved in transcriptional regulation or post-transcriptional regulation, according to their functional mechanisms. Among these mechanisms, splicing regulation and translation control are the two most important regulatory mechanisms of lncRNA in post-transcriptional regulation. In addition, lncRNA can also act as ceRNA to interact with miRNA to regulate downstream signaling pathways. LncRNA Gm2044 was used as a ceRNA to participate in spermatogenesis and estrogen regulation.

Infertility is a problem that has troubled people for a long time, and there is an urgent need to solve all kinds of infertility. LncRNA plays an important role in germ cell development, but research on lncRNA as a drug therapy is still in its infancy. There are a wide variety of lncRNAs that are expected to become potential targets for gene-drug therapy. Targeting one or more lncRNAs alone may be the most promising method for the treatment of infertility. At the same time, the lncRNA-miRNA-mRNA regulatory network also provides a new perspective for the treatment of various types of infertility.

In this review, we summarize recent findings on lncRNA Gm 2044, from the discovery of lncRNA Gm2044 to the biological functions it plays in the testis and ovary. LncRNA Gm2044 is highly expressed in specific stages of meiosis of female and male gametes, and can interact with a variety of miRNAs and transcription factors to participate in spermatogenesis or estrogen synthesis in follicles. LncRNA Gm2044 is also involved in the progression of NOA and PCOS, which provides a new target for the treatment of infertility. Whether lncRNA Gm2044 plays a role in other infertility or molecular signaling pathways remains to be investigated.

The differentially expressed genes *Tph1*, *Sost*, and *Zp3* found in lncRNA Gm2044 knockout mice are also related to follicle development (Hu et al., 2023). It is believed that with the elucidation of the functional mechanism of these genes in germ cells, a new regulatory network composed of lncRNA may be discovered, providing new ideas for the treatment of diseases of the reproductive system.
