# Corrigendum: Mechanism of LncRNA Gm2044 in germ cell development

**DOI:** 10.3389/fcell.2024.1485712

**Published:** 2024-09-11

**Authors:** Qinran Zhu, Junpei Sun, Chuangchuang An, Xin Li, Shumin Xu, Yutong He, Xinyi Zhang, Lei Liu, Ke Hu, Meng Liang

**Affiliations:** ^1^ School of Life Science, Bengbu Medical University, Bengbu, China; ^2^ First Affiliated Hospital, Bengbu Medical University, Bengbu, China

**Keywords:** lncRNA, spermatogenesis, estrogen, ovaries, miRNA

In the published article, the reference for “Hu et al., 2021” was incorrectly written as “Hu, K., He, C., Sun, X., Li, L., Xu, Y., Zhang, K., et al. (2021). Correction to: integrated study of circRNA, lncRNA, miRNA, and mRNA networks in mediating the effects of testicular heat exposure. Cell Tissue Res. 386 (1), 209. doi:10.1007/s00441-021-03480-1.” It should be “Hu, K., He, C., Sun, X., Li, L., Xu, Y., Zhang, K., et al. (2021). Integrated study of circRNA, lncRNA, miRNA, and mRNA networks in mediating the effects of testicular heat exposure. Cell Tissue Res. 386(1):127-143. doi: 10.1007/s00441-021-03474-z”.

In the published article, the reference for “Hu et al., 2023” was incorrectly written as “Hu, K., Wang, C., Xu, Y., Li, F., Han, X., Song, C., et al. (2023). Correction: interaction of lncRNA Gm2044 and EEF2 promotes estradiol synthesis in ovarian follicular granulosa cells. J. ovarian Res. 16 (1). doi:10.1186/S13048-023-01287-Y.” It should be “Hu, K., Wang, C., Xu, Y., Li, F., Han, X., Song, C., et al. (2023). Interaction of lncRNA Gm2044 and EEF2 promotes estradiol synthesis in ovarian follicular granulosa cells. J Ovarian Res. 16(1):171. doi: 10.1186/s13048-023-01232-z”.

In the published article, the name of one of the authors was incorrectly spelled in the reference for Hu et al., 2019 as “W, H.” It should be “Wang, H”.

The authors apologize for these errors and state that this does not change the scientific conclusions of the article in any way. The original article has been updated.

